# How Radiomics Can Improve Breast Cancer Diagnosis and Treatment

**DOI:** 10.3390/jcm12041372

**Published:** 2023-02-09

**Authors:** Filippo Pesapane, Paolo De Marco, Anna Rapino, Eleonora Lombardo, Luca Nicosia, Priyan Tantrige, Anna Rotili, Anna Carla Bozzini, Silvia Penco, Valeria Dominelli, Chiara Trentin, Federica Ferrari, Mariagiorgia Farina, Lorenza Meneghetti, Antuono Latronico, Francesca Abbate, Daniela Origgi, Gianpaolo Carrafiello, Enrico Cassano

**Affiliations:** 1 Breast Imaging Division, IEO European Institute of Oncology IRCCS, 20141 Milan, Italy; 2 Medical Physics Unit, IEO European Institute of Oncology IRCCS, 20141 Milan, Italy; 3 Postgraduation School in Radiodiagnostics, University of Milan, 20122 Milan, Italy; 4 UOC of Diagnostic Imaging, Policlinico Tor Vergata University, 00133 Rome, Italy; 5 Department of Radiology, King’s College Hospital NHS Foundation Trust, London SE5 9RS, UK; 6 Department of Radiology, IRCCS Foundation Ca’ Granda Ospedale Maggiore Policlinico, 20122 Milan, Italy; 7 Department of Health Sciences, University of Milan, 20122 Milan, Italy

**Keywords:** breast cancer, radiomics, medicinal imaging, personalized medicine, quantitative biomarkers, artificial intelligence

## Abstract

Recent technological advances in the field of artificial intelligence hold promise in addressing medical challenges in breast cancer care, such as early diagnosis, cancer subtype determination and molecular profiling, prediction of lymph node metastases, and prognostication of treatment response and probability of recurrence. Radiomics is a quantitative approach to medical imaging, which aims to enhance the existing data available to clinicians by means of advanced mathematical analysis using artificial intelligence. Various published studies from different fields in imaging have highlighted the potential of radiomics to enhance clinical decision making. In this review, we describe the evolution of AI in breast imaging and its frontiers, focusing on handcrafted and deep learning radiomics. We present a typical workflow of a radiomics analysis and a practical “how-to” guide. Finally, we summarize the methodology and implementation of radiomics in breast cancer, based on the most recent scientific literature to help researchers and clinicians gain fundamental knowledge of this emerging technology. Alongside this, we discuss the current limitations of radiomics and challenges of integration into clinical practice with conceptual consistency, data curation, technical reproducibility, adequate accuracy, and clinical translation. The incorporation of radiomics with clinical, histopathological, and genomic information will enable physicians to move forward to a higher level of personalized management of patients with breast cancer.

## 1. Introduction

**Summary**: this review summarizes the state of the art of radiomics in breast imaging and provide clinicians and researchers with the basis for a practical approach to this emerging field.

**Key points**:

- The current limitations in the diagnosis of breast cancer lie in not detecting valuable prognostic and predictive information inherent in the heterogeneity of this disease;

- Thanks to the advancement in AI technologies, radiomics can extract qualitative and quantitative information from images that can support clinicians in the management of patient with breast cancer;

- Radiomics showed valuable applications in breast imaging distinguishing between malignant and benign lesions, assessing the tumour subtype and its grade, molecular expressions, and predict response to therapy and the risk of recurrence.

Breast cancer (BC) is the most diagnosed tumour (excluding skin cancers), with an increasing incidence, and is the second leading cause of death from malignancy among females worldwide [1]. Though concerns regarding early detection and accurate diagnosis have been raised, continued efforts are required towards application of precision medicine in BC.

**Figure 1:**
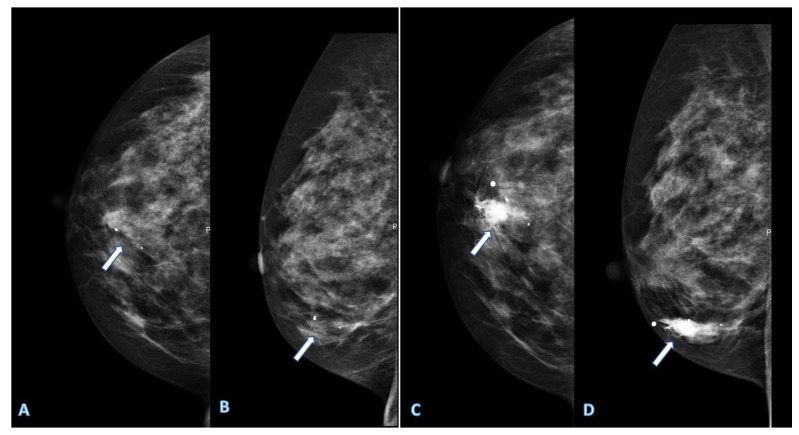
Digital mammography of a right breast shows some calcifications in an area (arrows) with increased radiopacity in cranio-caudal (**A**) and medio-lateral (**B**) mammograms. Contrast enhanced mammography shows an increased enhancement in the same area (**C**,**D**), which is suspicious for breast cancer.

**Figure 2:**
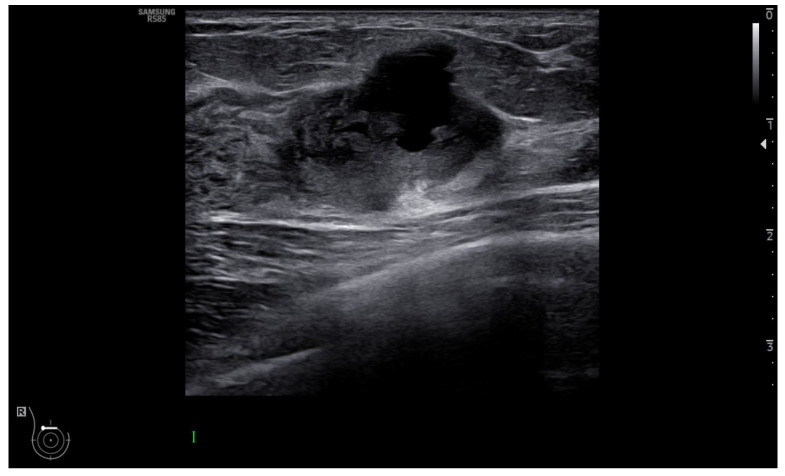
Ultrasound shows a heterogeneous hypoechoic mass lesion with small lobulations located in the upper quadrant of the left breast, highly suspicious for breast cancer.

**Figure 3:**
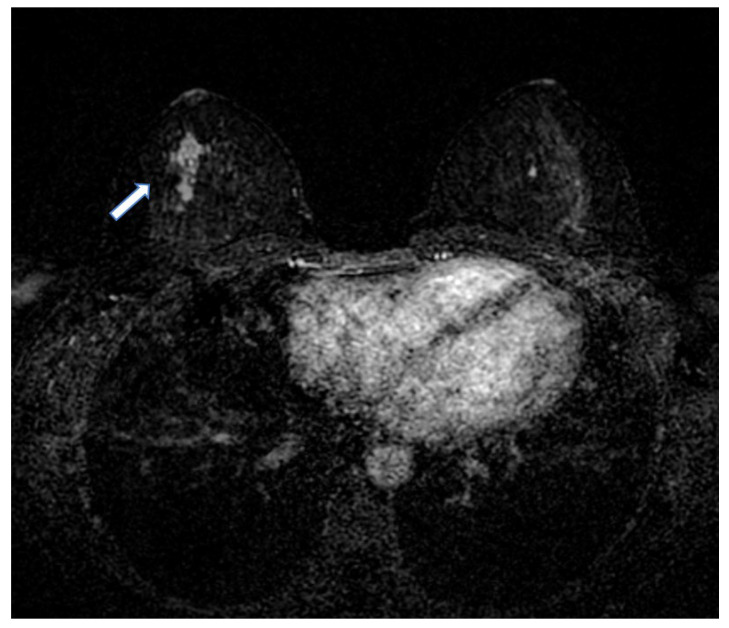
Breast MRI shows an enhancement (arrow) in the right breast suspicious for breast cancer.

Currently, the diagnosis of early invasive BC relies on radiological evaluation; essentially, based on mammography (with or without contrast) (Figure 1), breast ultrasound (US) (Figure 2) and contrast-enhanced magnetic resonance imaging (MRI) (Figure 3), supplemented by pathological confirmation of malignancy on radiologically obtained tissue samples [2,3]. However, such diagnostic approach has major limitations. Firstly, the sensitivity and positive predictive value are suboptimal [4]; secondly, biopsy is invasive and uncomfortable for women; thirdly, there is a long turnaround time for test results [5]. Moreover, BC is a heterogeneous disease with a significant chance that some of its features, including significant characteristics, remain undetected, meaning that valuable prognostic and predictive information can be missed. Due to such heterogeneity and dynamic tumour biology, indications for re-biopsy are increasing [6]. This is pertinent in the current era of personalised medicine, which relies on early diagnosis of disease, in a patient with specific characteristics, and the subsequent individually tailored treatments, aiming to deliver the right treatment to the right patient at the right time. Accordingly, the ultimate goals of the modern breast imaging are to detect BC as early as possible, then to classify the lesion and predict its clinical course and its biological aggressiveness to optimise treatment in a specific patient [7,8].

Continued exponential growth of medical imaging has led to an advancement in radiomics, which provides countless quantitative biomarkers extracted from modern diagnostic images, including detailed tumoral characterization of BC [9]. In particular, recent technological advances in the field of artificial intelligence (AI) applied to image analysis, via software based on machine learning (ML) and deep learning (DL), hold promise in addressing medical challenges in cancer detection, treatment assessment, prediction of treatment response and monitoring of disease progression [9,10,11,12,13,14,15,16,17,18]. In the current breast imaging practice, evaluation of BC is largely qualitative, including subjective evaluations such as tumour morphology, enhancement curves, and anatomic relationship to the surrounding tissues. However, to achieve the goal of personalised medicine, a quantitative evaluation is also required [19,20]. Therefore, radiomics is an emerging field of extreme interest, dealing with quantitative evaluation of images and extraction of designated features [21,22,23]. Data derived from a radiomics investigation, such as intensity, shape, texture, and wavelength [22,24,25,26,27], can be input to ML or DL algorithms, providing information to differentiate malignant and benign tumours, assess cancer genetics, predict treatment response, and contribute to more robust models that combine multidisciplinary information [11,16,22,28,29,30].

This review discusses the state of the art of radiomics, in both research and clinical applications, and its role in achieving personalised management in patients with BC. This paper will also try to demystify radiomics for clinicians by illustrating its limitations and challenges, as well as the opportunities it provides as a decision-support tool in cancer management.

## 2. Strengths and Limitations of Current Breast Imaging Techniques

Breast cancer screening with mammography has significantly lowered the breast cancer mortality rate; however, its sensitivity is limited in dense breasts. In this situation, ultrasound is a helpful imaging modality to examine the glandular and ductal components, investigate the axillary lymph nodes, and avoid the use of ionizing radiation. For these reasons, ultrasound is the primary diagnostic imaging technique in women under the age of 40.

Dynamic contrast-enhanced (DCE) MRI and contrast-enhanced spectral mammography (CESM) offer the advantage of adding a functional evaluation to the morphological data through the administration of contrast medium that exploits neo-angiogenesis occurring in tumoral lesions [31,32,33,34]. Nowadays, MRI is accepted as the most sensitive imaging technique for detecting and staging breast cancer and is of great help in the study of particularly complex breasts, or of patients with an elevated hereditary–familial risk of developing breast cancer [35]. With a similar performance to MRI, CESM can also visualize microcalcifications and possibly reduce the rate of false-positive findings and benign biopsies [32,36].

Radiomics features of BC can be extracted from MRI [37,38,39,40,41,42,43,44,45,46,47,48,49,50,51,52,53], US [54], PET/CT [55,56], CEM and Mx and tomosynthesis images [51,57]. The most-used imaging biomarkers in breast cancer are those derived from perfusion and diffusion imaging. The perfusion imaging is both an MRI and CEM technique based on intravenously administered contrast agents, that enables spatially resolved quantification of the hemodynamic status of tissue. The first assumption is that the contrast media administration induces time-dependent changes in tissue signal, which can be monitored by the dynamic acquisition of images before, during and after contrast media injection. The second postulation is that pathological tissues have different hemodynamic properties from normal tissues, that can be depicted by perfusion biomarkers [58]. Diffusion-weighted imaging (DWI) is an MRI technique that uses quantitative estimation of random motion of water molecules as a surrogate for tissue characterisation, and it can be successfully employed in oncology to detect pathological structural changes [27,28,58].

Image guided percutaneous breast lesion biopsy can be performed using mammography, US, or MRI visualisation. The choice of the biopsy method is generally dictated by the imaging characteristics of the lesion, patient factors, and modality availability. 

Compared to surgical biopsy, percutaneous imaging-guided biopsy is less invasive, faster, more cosmetically satisfactory, and the recovery time is shorter [19,59,60]. In the case of vacuum-assisted biopsies, the procedure may also be curative as small lesions can be removed completely [61]. All these percutaneous procedures also allow the placement of a harmless lesion marker, which is useful for the recognition of the target lesion during surgery or after neoadjuvant therapy (NAT—chemotherapy performed before surgery). The NAT may lead to resolution of the tumour without detectable mass, and lesion markers may be helpful to confirm complete treatment response [16,18,19].

Although percutaneous image-guided procedures are generally safe and highly accurate, there is still the risk of infrequent (<1 in 1000) complications, which include pain, bleeding, post procedural infections, and, rarely, pneumothorax and pseudoaneurysms [62]. In addition to relatively easily managed complications of haematomas and infections, some complications such as an arterial pseudoaneurysm or a pneumothorax, may be more difficult to manage. Although controversial, neoplastic seeding may occur, quoted in the literature in up to 1–2 cases per 1000 US-guided biopsies [63]. Moreover, as percutaneous biopsies only sample a part of the lesion, the examination may not be conclusive. In fact, in 3–9% of cases the malignant potential of lesions is uncertain, and up to 33% of these manifest eventually as clinically significant malignancy [63].

## 3. What Radiomics Is and How it Works in Breast Imaging Workflow

Radiomics assumes that radiological images contain more information than is visible to human eyes. Thus, radiomics is a translational field of research aiming to find associations between qualitative and quantitative information extracted from medical imaging and clinical data, to support evidence-based clinical decision making [9]. The extraction of quantitative features from radiological images allows the creation of high-dimensional data with clinical data. This is followed by data mining to extract valuable information for decision support models.

Radiomic workflow involves the following steps:

1. Images acquisition;
2. Images segmentation;
3. Features extraction;
4. Features selection;
5. Model construction.

**Figure 4:**
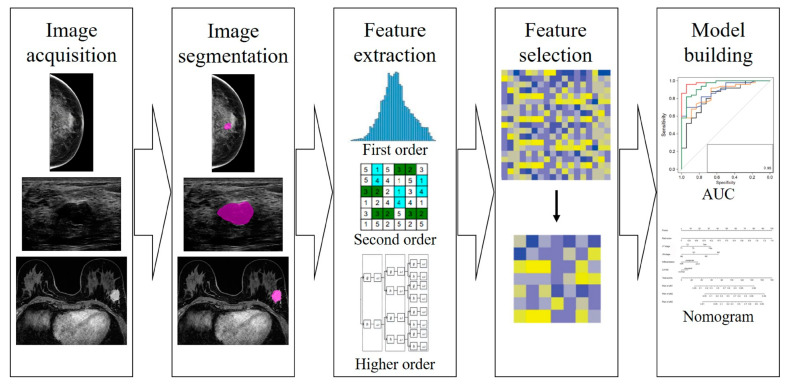
Different steps of radiomics workflow in breast imaging.

Such steps are shown in Figure 4.

In breast imaging, the pixel value of MRI, US, and mammography, does not express physical properties of the tissue, such as Hounsfield Unit in computed tomography, but it is dependent on acquisition parameters.

In addition, MRI signal may change even with the same reconstruction parameters for two consecutive acquisitions, while US acquisition is also operator dependent [64,65]. To overcome this limitation, it is a good practice to acquire all imaging data using the same device and imaging parameters to ensure the stability of the features and record the parameters meticulously in order to facilitate the reproducibility of the study.

When this approach is not feasible, harmonization is mandatory to ensure robustness of features and generalizability of the model obtained [66].

Image segmentation with delineation of Regions of Interest (ROI) is a crucial part of the radiomic workflow. ROIs limit the area of analysis, and their delineation can be obtained manually, in a semi-automatic or fully automatic way.

Manual segmentation may introduce observer-bias, as studies have shown that many radiomic features are not robust against intra- and inter-observer variations concerning ROI delineation [67]. Consequently, studies using manual image segmentation with manual correction should perform assessments of intra- and inter-observer reproducibility of the derived radiomic features and exclude non-reproducible features from further analyses [68].

Semi-automatic segmentation may potentially reduce these issues. It has been demonstrated to work well for relatively homogeneous lesions; however, inhomogeneous lesions with poorly defined boundaries require intensive user correction [65].

Fully automatic segmentation based on DL networks is rapidly emerging, and many different algorithms have already been trained for image segmentation tasks of various organs. Such algorithms need ad hoc training and quality control, with manually contoured images as reference [9,69]. Generalizability of trained algorithms, however, is a major drawback, given that applying those algorithms on a different dataset often results in complete failure [9,68].

Feature extraction is the calculation of mathematical expressions used to quantify characteristics of the grey levels within the ROIs. Since many ways and formulas exist to calculate those features, adherence to the Image Biomarker Standardization Initiative guidelines is recommended.

Features can be extracted either directly from the images or after applying different filters, and they are usually categorised into the following subgroups:

Shape features describe the shape of the traced ROI and its geometric properties such as volume, maximum diameter along different orthogonal directions, maximum surface, tumour compactness, and sphericity.

First-order statistics features describe the distribution of individual voxel values without concern for spatial relationships. These are histogram-based properties reporting the mean, median, maximum, minimum values of the voxel intensities on the image, as well as their skewness (asymmetry), kurtosis (flatness), uniformity, and randomness (entropy).

Second-order statistics features include the so-called textural features, which are obtained by calculating the statistical inter-relationships between neighbouring voxels. They provide a measure of the spatial arrangement of the voxel intensities and, hence, of intra-lesion heterogeneity.

Higher-order statistics features are obtained by statistical methods after applying filters or mathematical transforms to the images.

The subsequent step involves feature selection, in order to exclude all the features that are either not reproducible or not strongly related to the outcome.

Selection can be made through statistical methods or through ML methods. The first starts from all the features provided by the calculation tool and performs a preliminary analysis to select the most repeatable and reproducible parameters and to subsequently reduce them by correlation and redundancy analysis [70].

Alternatively, ML techniques, underlying the idea that computers may learn from past examples and detect hard-to-discern patterns from large and complex data sets, may lead to the selection of appropriate features [71].

Finally, the remaining, non-correlated and highly relevant features can be used as input to the model for the respective classification task (e.g., discriminate between malignant or benign lesion).

Models are usually built by splitting the dataset into training and test sets, and most robust models are usually validated using a totally external dataset, to ensure generalizability of the obtained results [72].

## 4. The Role of Artificial Intelligence and Big Data in Radiomics

Mammography was one of the first imaging modalities to incorporate AI techniques, beginning with traditional computer-aided detection (CAD) [73]. CAD systems for mammography have been available for over a decade, meaning that the application of more recent ML and DL techniques to mammography has an existing benchmark for comparison [73].

Since then, significant advances in imaging analysis and the development of high-throughput methods have facilitated the rapid and simultaneous extraction and correlation of multiple imaging parameters [74].

Artificial intelligence is often associated with radiomics, given that it can be exploited in different steps of the radiomic workflow. For instance, AI can perform the task of image segmentation, before radiomic features are extracted. However, this approach is not often used, because with DL, the steps of image segmentation, feature extraction and classification are usually performed as a unique task [75].

When handcrafted features are extracted, ML algorithms, such as random forests, neural networks, linear regression, logistic regression, least absolute shrinkage, and selection operator, can help in feature selection before building the model [76].

AI studies must pass through rigorous validation steps including defining the imaging data sets (training, validation, and test sets), defining the ‘ground truth’ reference standard, having a detailed description of the training approach and metrics of model performance, and having validation or testing of the algorithm with external data. Three independent data sets (training, validation, and test sets) are needed: first, the AI algorithms are trained on an initial set of images according to a reference standard; second, the final algorithm is validated on a separate set of images; third, an external set of images is used to report the final statistical results of the AI algorithm [77]. 

AI methods can relate imaging-based characteristics to clinical, histopathology, or genomic data, contributing to precision medicine [9].

Moreover, currently unknown correlations between observed phenotypes and genotypes may be discovered through the mappings between imaging data and genomic data, providing possibilities to improve early detection and better management of the disease [8,21,22,23].

## 5. Recent Radiomics’ Application in Breast Cancer Care

The recently developed AI algorithms on vast amounts of imaging data has led to satisfactory models for the application of radiomics in breast cancer care, and even the patients approve the introduction of AI in clinical practice although only as a support to radiologist, and not in substitution thereof [78]. Indeed, radiomics already showed valuable applications in breast imaging practice: it may distinguish between malignant and benign lesions, assess the tumour subtype and its grade, assess the molecular expressions, and predict response to therapy and the risk of recurrence [5,9,16,17,18,20]. With the ability to infer the molecular profile of the tumour, a specific mutation or genotype, or even defining treatment possibilities and prognosis in BC patients, radiomics data may substitute physical breast biopsies in the near future [9,17,20].

Although radiomics and radio-genomics have great potentialities and offer some promising applications for personalised medicine [21,22], independent validation datasets are still needed to confirm the diagnostic and prognostic value of such technologies. They still need time before playing a significant practical role in cancer research and even more time to reach clinical practice. This is essentially due to the limitations of the available big data, which often lacks complete characterisation of the patients, poor integration of individual datasets, and a widespread misperception about their use and sharing [79].

The following are the updated state of the art of radiomics’ application in breast cancer care.

### 5.1. Radiomics as a Virtual Biopsy in Breast Cancer Diagnosis and Classification

The early detection and characterization of BC is crucial to improve outcomes in women because small non-metastatic disease can be effectively treated with curative intent [2,80,81]. The diagnosis of breast cancer currently relies on radiological and clinical evaluation, confirmed by histopathological examination. However, such an approach has limitations of suboptimal sensitivity, long turnaround time for test results, the invasiveness of the procedure, and the risk that some features of target lesions may remain undetected, requiring repeat biopsy.

Radiomics, through the extraction of quantitative peculiar features of BC from imaging data, may identify diagnostic information of breast cancer, potentially reducing the need for invasive biopsies, and facilitating an approach that is as personalised as possible for each patient. From the perspective of truly personalised management of breast cancer, based on early diagnosis and individually tailored treatments, radiomics is rising as a means to obtain information from diagnosis to molecular profiling, and treatment response assessment indeed, without the need of a physically biopsied tissue sample. 

Zhou et al. [51] used 99 texture and histogram parameters from 133 patients who underwent DCE-MRI to differentiate between benign and malignant BC with 91% of accuracy. Xie et al. [82] analysed radiomics features extracted from 134 BC with similar accuracy, comparing triple negative breast cancer (TNBC) to the non-TNBC at breast MRI.

In 2018, a retrospective study [83] analysed unenhanced DWI-based radiomics to determine the malignant nature of suspicious breast lesions detected on screening mammography, decreasing the false-positive results in lesions classified as BI-RADS 4 or 5 at screening mammography while retaining sensitivity greater than 98%.

Li et al. [84] analysed the radiomic features from mammography in 182 patients (106 malignant and 76 benign), showing that the performance of the combined lesion and parenchyma classifier in the differentiation of malignant and benign findings was better than that which only used the lesion features.

In 2019, a sub-study of a multi-centre and prospective study leaded by Tagliafico et al. [85] applied a radiomics approach to tomosynthesis for the first time to differentiate normal from malignant breast tissue in patients with dense breasts in a small number of 40 patients, showing encouraging results.

Luo et al. [86] used radiomics features extracted from breast US of 315 patients to discriminate benign from malignant lesions.

In 2017, Fan et al. [43] analysed a combined model of DCE-MRI-based radiomics features and clinical information to predict molecular subtype of BC, with 0.87 of AUC value. Similar promising results were reported in the 2019 by Xie et al. [87] with texture features extracted from unenhanced breast MRI, and the 2020 by Demircioglu et al. [88], on a population of 98 women, showed the usability of a simplified and rapid approach to tumour for MRI-based decoding and phenotyping of BC. 

Despite such encouraging results, radiomics technology is still not ready to substitute tissue biopsy in the near feature, and even then, they will require the aid of other parameters to be correctly interpreted and acted upon [9,20].

### 5.2. Prediction of Response to Neoadjuvant Chemotherapy 

In the last decade, NAT has been increasingly used to treat operable BC, and it is associated with a favourable treatment response in 30% of women with aggressive BC, and decreases the rate of recurrence by up to 50% [2].

The achievement of pathological complete response (pCR) is a powerful prognostic factor for long-term outcome, and it is considered as the only currently validated biomarker of survival. However, it can only be assessed at surgery so far [89,90]. Therefore, radiomics may allow a non-invasive and earlier detection of treatment resistant lesions, to avoid the unnecessary toxicity of chemotherapy, and delayed access to other, potentially effective, therapies. 

Several recent studies proposed prediction models of pCR to NAT in BC based on MRI [16,20,89,91,92,93], and valuating the pCR prediction by the extraction of radiomics features from pre-NAT breast MRI, obtaining statistically significant results [9,16,18,50,94,95,96,97].

In 2020, Choudhery et al. [97] used morphological and three-dimensional textural features to predict the molecular subtype and the pCR in 259 BC patients treated with NAT, showing significant association with pCR and residual cancer burden in BC. In 2017, Braman et al. [96] evaluated radiomic features based of both peri- and intra-tumoral regions on pre-treatment DCE-MRI to predict the pCR to NAT in 117 BC patients, demonstrating that peri-tumoral radiomics contributed to the successful prediction of the pCR of BC patients, yielding a maximum AUC of 0.74 within the testing set.

Previously, other authors [43,50,94,95,98] showed that quantitative analyses of radiomic features from pre-treatment breast DCE-MRI data in BC patients could be used as valuable image markers that are associated with pCR to NAT.

In the above-mentioned studies, DCE had been used more frequently than DWI to extract radiomics feature as it can provide the tumour’s kinetic characteristics of the contrast agent by producing pharmacokinetic maps. 

In a multicentre study, Liu et al. [91] utilized multiple MRI sequences, including DWI, to predict pCR to NAT in BC patients. A total of 586 patients were enrolled, and a radiomic score was calculated using 13,950 features. Quantitative analyses extracted from MRI provide a promising tool for predicting tumour response in patients with advanced BC and show the potential and practical value in the clinic.

In 2021, our team performed a retrospective mono-centric study with the aim to assess radiomics with MRI for the early prediction of pCR in 83 BC patients undergoing NAT, investigating the correlation between pre-NAT radiomics with DCE-MRI features and disease-free survival (DFS), and the correlation between post-NAT (residual BC tissue) radiomics features and DFS [18]. Using 136 representative radiomics features selected through cluster analysis from the 1037 extracted features, a radiomic score was calculated to predict the response to NAT, with AUC of 0.64. After combining the clinical, biological and radiomics models, the AUC was 0.83, showing that MRI-based radiomic features slightly improved the pre-treatment prediction of pCR to NAT, in addiction to biological characteristics. The identification of the non-pCR patients in high-risk subgroups (defined by radiomics features), if confirmed on larger cohorts, could be helpful to identify such patients, to avoid unnecessary treatment.

### 5.3. Radiomics for Predicting Lymph Node Metastasis

Axillary lymph node (ALN) status is among the most important breast cancer prognostic factors [99,100]. Radiomics showed encouraging results in predicting the presence of ALN metastasis. Accordingly, several studies aimed to develop and validate radiomic nomograms as new tools based on radiomic signatures and clinical-pathologic risk factors to stratify patients more precisely in risk categories [101,102,103,104].

Radiomic nomograms proved to be reliable either when radiomic signatures were extracted from mammography (AUC up to 0.88) [101], CEM (AUC up to 0.79) [104] and MRI (AUC 0.89) [103]. In the latter, Yu et al. also assessed preoperative identification of ALN metastases and individual DFS in patients with early-stage BC [103].

More recently, the same team [105] implemented the approach with a multi-omics signature incorporating MRI multi-sequence key radiomic features of ALN and tumour regions with clinicopathologic characteristics and molecular subtype.

**Figure 5:**
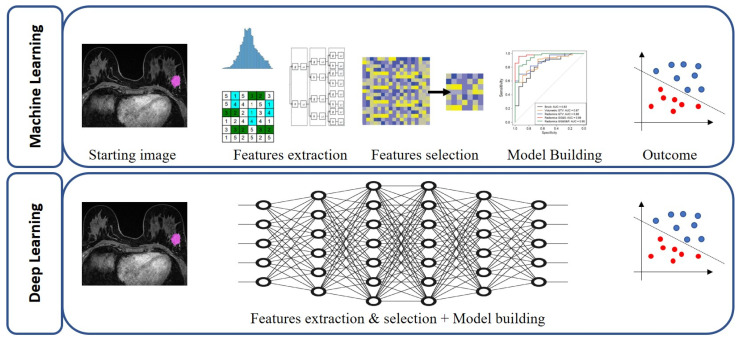
Difference between machine learning (ML) approach and deep learning (DL) approach, in which the steps of feature extraction, selection and classification are performed as a unique task.

Recurring limitations of the above-mentioned studies were reliance on the retrospective design of the studies themselves, the heterogeneity of imaging parameters, and the absence of standardization in the extraction and developing of radiomic features, highlighting the common issues of radiomics studies. Cattell et al. [106] aimed to compare the generalizability of conventional radiomics vs. deep learning based features (using DCE-MRI) in an independent test set with dissimilar resolution, while developing a prediction model for preoperative prediction of SLN metastases. Interestingly, the features based on DL outperformed the conventional radiomic model accuracy, particularly in the independent testing set of dissimilar resolution, indicating that such features can ultimately result in a more generalizable model. Figure 5 shows the difference between ML and DL approach, in which the steps of feature extraction, selection and classification are performed as a unique task.

### 5.4. Radiomics for Predicting Breast Cancer Recurrence

Recurrence is the principal cause of breast cancer-related death [107]. Radiomics may play a role in the prediction of the risk of BC recurrence with the double potential benefits to reduce overtreatment in the low-risk patients and to reduce the undertreatment in the high-risk patients with BC. 

Even for the prediction of BC recurrence, the most used imaging modality in the field of radiomics is the breast MRI. Three recent studies [53,108,109] found a statistically significant correlation between the risk of recurrence and radiomic features extracted from pre-treatment breast DCE-MRI in patients with invasive BC. In 2018, Park et al. [53] evaluated 294 MRI and drawn ROIs on entire tumour volume from each of the four distinct dynamic-series resulting in a radiomic signature which was significantly associated with worse DFS. In 2020, Chitalia et al. [108] analysed the MRI of 95 BC patients, extracting radiomics features from the entire tumour volume using only the first and second post- contrast images: 22 radiomics features were selected to create a tumour heterogeneity index that identified phenotypes of low, medium, and high intra-tumour heterogeneity with statistically significant differences in 10-year recurrence-free survival across phenotypes. In 2019, Mazurowski et al. [109] evaluated radiomics features of the MRI of 892 BC patients and found tumour size, textural and volumetric measures related to the enhancement had the strongest association with DFS in the univariate analysis. Most of these variables were also independently prognostic of outcomes in a multivariate analysis, after controlling for clinical and pathologic variables. Moreover, the authors also evaluated the prognostic value of each of the selected variables for specific clinically relevant subsets of patients (e.g., patients with ER/PR positive tumours, who underwent NAT or received hormonal therapy), and this increases their applicability in clinical settings.

A recent retrospective study [110] demonstrated that combining MRI radiomic features of BCs with MRI radiomic features of normal parenchyma from the contralateral breast, helps in the prediction of BC recurrence, suggesting that the underlying breast environment may contribute to recurrence more than the cancer biology of the tumour alone.

In 2020, Koh et al. found an association between MRI radiomics features and systemic recurrence in patients with TNBC. They selected 32 features on the second phase of DCE-MRI of patients with TNBC before any treatment. Their radiomics model, obtained by combining the Rad score with clinical and pathologic data, better predicted systemic recurrence than that of the clinical model alone. However, when they tried to validate the results with a different MRI scanner, the external validation did not show the radiomics model to be superior to the clinical model.

In addition to MRI, even mammography and US were used to extract radiomics features for prediction of histological findings. 

In 2021 Xiong et al. [111] developed the first radiomics signature based on US to predict DFS in women with invasive BC. Their radiomics nomogram proved to be superior to the clinicopathological nomogram in terms of clinical usefulness. However, a limit of this US radiomic signature was that it was not reliable for DFS prediction when different sonographic platforms were used. 

In a multicentric study, Yu et al. [112] developed and validated a recurrence risk model based on radiomics features from US for TNBC patients. The radiomics model, incorporating a radiomics signature and three prognostic variables, had a better diagnostic performance than that of a radiomics signature and clinicopathological model when used alone.

In the same year, Dasgupta et al. [113] conducted a prospective study to investigate the role of pre-treatment quantitative US radiomics in predicting recurrence for patients with locally advanced BC. They obtained 95 radiomics features including spectral features, texture, and texture-derivatives ones that have been demonstrated to be related to tissue microstructural elastic properties. These parameters were selected using the support vector machine-based model that demonstrated an accuracy of 82% to identify BC patients developing recurrence. 

Concerning mammography, in 2021, Mao et al. [114] developed a mammography-based radiomics model for predicting the risk of BC: the multivariate logistic regression model including radiomics signature and clinical risk factors (tumour grade and HER 2) showed good performance yielding AUC of 0.92 in the training set.

Overall, these studies have shown that that adding radiomics to the standard radiological workflow would improve the prognostic value of breast imaging. 

## 6. Future Trends and Modern Perspectives

Precise identification of cancer subtype and biomarkers of tumour immune biology play an increasingly important role in prognostication and treatment selection. The oncological drive towards multifocal biopsy of heterogenous lesions, multisite biopsy in metastatic disease, and resampling following therapy calls for non-invasive alternatives [115]. Fusion of data collated in The Cancer Imaging Archives (TCIA) and The Cancer Genome Atlas (TCGA), with increasingly capable radiomics workflows and the emergence of radiogenomics stands poised to provide the requisite solutions [116,117,118,119]. Non-invasive biomarkers for predicting response to specific treatments are being validated, and radiomics signatures are en route to support clinical practice. Innovation will be explosive, and radiologists are the essential partners in testing the translational applications of these technologies [20].

## 7. Conclusions

Data from the above-mentioned studies show the advancement in AI technologies in healthcare and support the potential utility of radiomics analysis in determining breast cancer biomarker from diagnostic imaging. However, standardization and transparency across all stages of the radiomics workflow and refinement of AI algorithms are required for improving reproducibility of radiomics analysis.

The assessment of clinical relevance and impact prior to study commencement, increased level of evidence using studies with large enough datasets and external validation, and its combination with established methods will help to move the field towards clinical implementation. As radiomics applications in breast cancer care include diagnosis, prognostication, and prediction of treatment response, a multidisciplinary collaboration among radiologists, data scientists and imaging scientists is demanded.

Multi-centre prospective oncological and radiological translational research using real-world heterogeneous datasets may develop radiomics techniques as the paradigm shifts towards minimally invasive techniques in the scenario of personalised medicine.

## Data Availability

No new data were created or analyzed in this study. Data sharing is not applicable to this article.

## Glossary

Diffusion Weighted Imaging (DWI)	MRI technique based on the random Brownian motion measure of water molecules within a voxel of tissue.
Machine learning	A branch of artificial intelligence involving use and development of computer systems that are able to learn and adapt without following explicit instructions, by using algorithms and statistical models to analyse and draw inferences from patterns in data.
Radiomic features	Characteristics of a tissue or a lesion that can be extracted in form of data.
ROI	acronym of Region of Interest, the portion of an image over which radiomics features are extracted. It can be delineated manually, in semi-automatic of fully automatic way.
Radiomic signature	Computational model which aims to address either unmet clinical needs, mostly in the field of oncologic imaging, as biomarkers, for example, or to compare radiomics performance with that of radiologists.
